# Literature-aided meta-analysis of microarray data: a compendium study on muscle development and disease

**DOI:** 10.1186/1471-2105-9-291

**Published:** 2008-06-24

**Authors:** Rob Jelier, Peter AC 't Hoen, Ellen Sterrenburg, Johan T den Dunnen, Gert-Jan B van Ommen, Jan A Kors, Barend Mons

**Affiliations:** 1 Department of Medical Informatics, Erasmus MC University Medical Center, Rotterdam, The Netherlands; 2 Department of Human Genetics, Leiden University Medical Center, Leiden, The Netherlands

## Abstract

**Background:**

Comparative analysis of expression microarray studies is difficult due to the large influence of technical factors on experimental outcome. Still, the identified differentially expressed genes may hint at the same biological processes. However, manually curated assignment of genes to biological processes, such as pursued by the Gene Ontology (GO) consortium, is incomplete and limited. We hypothesised that automatic association of genes with biological processes through thesaurus-controlled mining of Medline abstracts would be more effective. Therefore, we developed a novel algorithm (LAMA: Literature-Aided Meta-Analysis) to quantify the similarity between transcriptomics studies. We evaluated our algorithm on a large compendium of 102 microarray studies published in the field of muscle development and disease, and compared it to similarity measures based on gene overlap and over-representation of biological processes assigned by GO.

**Results:**

While the overlap in both genes and overrepresented GO-terms was poor, LAMA retrieved many more biologically meaningful links between studies, with substantially lower influence of technical factors. LAMA correctly grouped muscular dystrophy, regeneration and myositis studies, and linked patient and corresponding mouse model studies. LAMA also retrieves the connecting biological concepts. Among other new discoveries, we associated cullin proteins, a class of ubiquitinylation proteins, with genes down-regulated during muscle regeneration, whereas ubiquitinylation was previously reported to be activated during the inverse process: muscle atrophy.

**Conclusion:**

Our literature-based association analysis is capable of finding hidden common biological denominators in microarray studies, and circumvents the need for raw data analysis or curated gene annotation databases.

## Background

The comparative analysis of expression microarray studies can refine conclusions and interpretations from individual studies and can be used to identify previously uncharacterized parallels between studies [1,2]. However, such analyses are hampered by the large influences of biological variation between specimens (see e.g. Eid-Dor et al. [3]), and technical differences between the studies [4-9] on the identified differentially expressed genes. The varying technical factors include: differences in experimental procedures for the collection of the biological material and for RNA amplification and labeling [8], differences in sampling times and the DNA microarray platform used (see Kuo et al. [9] for a recent platform comparison), and the applied statistical analysis [6].

To overcome this hurdle, it has been suggested that studies should be compared at the level of perturbed biological processes [10,11]. This could be more robust as different genes may hint at the same process. To identify perturbed biological processes, methodologies have been developed in recent years by analysis of the correlated behaviour of groups of genes with a similar biological function [11-13]. A limitation when using these approaches is that to identify which genes share a biological function, we are currently largely dependent on the ontology-based annotation of genes in manually curated databases. Due to the labor intensive manual curation effort, these databases are necessarily highly focused and notoriously incomplete (see e.g. Khatri et al. [14]). The best known public databases are the Gene Ontology (GO) annotation project [15] for biological process, molecular function and cellular localization, and KEGG [16] for metabolic pathways.

In the present study we introduce an approach to compare expression profiling studies based on perturbed biological processes. Instead of using manually curated gene annotation databases we base our approach on gene associations automatically derived from literature. To identify gene associations, concept profiles are generated for all genes [17]. A concept profile is a weighted list of biological concepts that characterizes the set of documents associated to a gene. Subsequently concept profiles are compared to identify gene associations: pairs of genes strongly associated to the same biological concepts. Finally, DNA microarray datasets are compared based on the observed number of gene associations between the sets of differentially expressed genes. We call our approach LAMA (Literature-Aided Meta-Analysis).

We evaluate our methodology on a compendium of 102 DNA microarray studies published in the field of muscle development and disease, and compare it to analyses based on gene overlap and the classical group overrepresentation analysis. The compendium contains a very diverse set of datasets: patient versus control studies for different myopathies; studies in animal disease models and studies in cultured muscle cells. The studies were performed on 22 different microarray chip types, and in three different organisms: human, mouse and rat. The considerable influence of the statistical analysis on the identified differentially expressed genes [6], indicates that, ideally, a standardized statistical analysis should precede any comparison between datasets. Unfortunately, raw data is required for such an analysis and they are often unavailable (see also Larsson et al. [2]). Therefore, we relied on the reported lists of differentially expressed genes, which should be useful for initial comparisons of microarray studies [18,19] and, at least, were judged by the authors to be biologically relevant. In our evaluation, we first take a directed approach: we measured to which extent the approaches could reproduce a manual clustering of a selection of datasets. Second, we perform an exploratory clustering of all datasets, and characterize and interpret the identified clusters.

## Results

### Study selection and data retrieval

The 102 microarray datasets in the compendium are represented and annotated [see Additional file 1] and the data underlying the analyses is included [see Additional file 2]. The compendium was extracted from 53 publications and 6 in-house studies. The datasets include studies on myoblast differentiation as an *in vitro *model for muscle development and regeneration, studies on gene expression differences between different types of skeletal muscles, skeletal muscle disease (including induced muscular atrophy), the effect of exercise and ageing and the treatment with drugs, growth factors or lipid infusion. The compendium was limited to studies in human (N = 37), mouse (N = 51), and rat (N = 13), but included one study in monkey performed with a human DNA microarray platform. To allow for a direct comparison of datasets from different organisms, homologous genes were mapped to each other according to the NCBI's homologene database [20].

### Frequency of differential expression per gene

**Figure 1:**
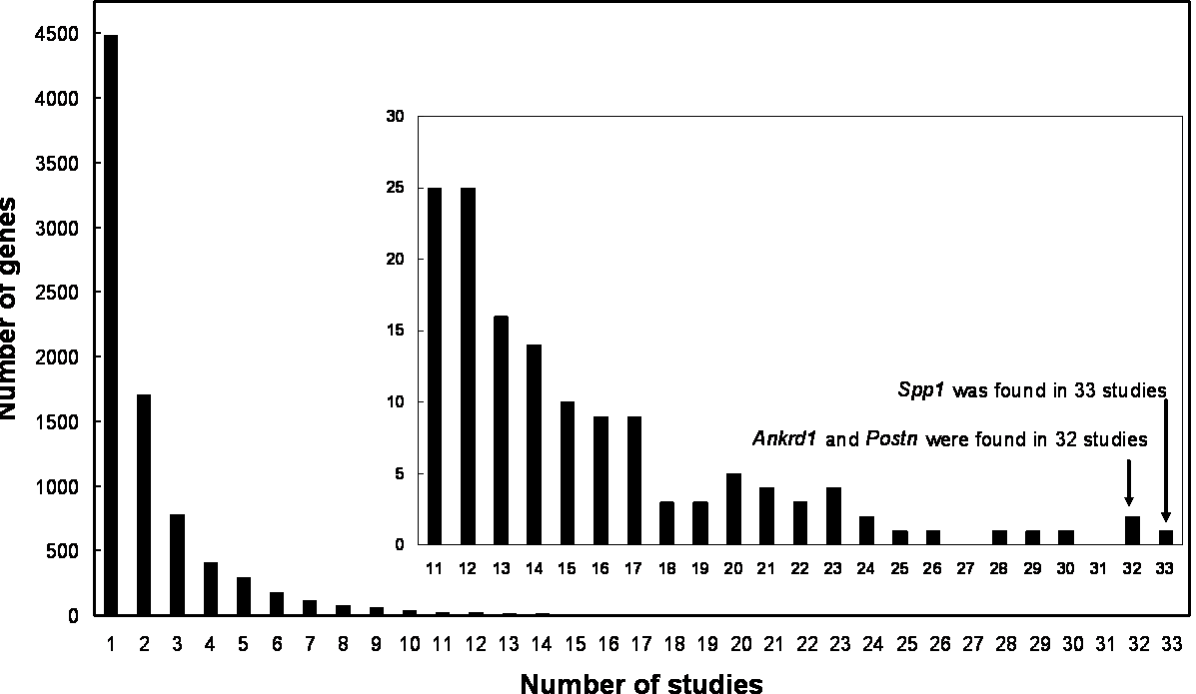
**Distribution of the number of microarray studies in which a gene was found differentially expressed**. A total of 102 studies was included with 8282 unique differentially expressed genes.

After mapping of species-specific Entrez Gene IDs to Homologene, 8282 unique genes were identified as differentially expressed in at least one microarray study. Figure 1 displays the distribution of the number of microarray studies in which a gene was found differentially expressed. The majority of genes (4486) was found differentially expressed in only a single study. The distribution implies that the overlap between studies is limited. Indeed, 84% of the genes occur in 3 or less studies, but they represent 54% of the total number of occurrences. *Spp1 *coding for osteopontin was the most frequently identified gene; it was found in 33 different studies described in 15 different papers coming from 8 different laboratories. *Spp1 *was upregulated in animal models for muscular dystrophy and in human polymyositis and dermatomyositis, and can be regarded as an early marker for muscle inflammation [21,22]. Conversely, it was found downregulated in presymptomatic *mdx *mice and during atrophy. *Ankrd1 *and *Postn *were found in 32 different studies. Judged from the studies in which *Ankrd1 *is differentially expressed, *Ankrd1 *could be the most robust marker for muscular dystrophy with ongoing regeneration. *Postn *(periostin) was differentially expressed in many of the studies in which *Spp1 *was found. A similar co-regulation was found in the heart and vasculature [23,24], and both factors are involved in tissue remodelling [22,25,26].

### Pair-wise analysis of similarity between DNA microarray datasets

First, we compared the DNA microarray studies to each other based on gene identity. For every study, the genes interrogated by the DNA microarray platform were separated into three categories: upregulated, downregulated and not up- or downregulated. With the kappa statistic [27] we measured the chance-corrected level of agreement in the three categories between two studies. By performing a kappa statistic based test [28] we found that of the 5151 possible dataset pairs only 307 (6%) have an above chance level of agreement (p < 0.05). This is in line with our conclusion of limited overlap in the previous section. Second, our LAMA method found significant associations (p < 0.05) between 2732 (53%) pairs of studies, which indicates that considerably more similarities between datasets are identified with our text-derived gene associations than based on gene list overlap.

Third, we compared datasets based on over-represented GO codes. We tested over-representation of biological processes in the up and down lists of our datasets separately with a cutoff of p < 0.05 (hypergeometric test). Subsequently, we evaluated the similarity between datasets with the kappa measure: Only 18% of the dataset pairs had a significant overlap (p < 0.05), whereas 34% of scores was very low (<0). The used GO over-representation p-value cutoff is overly permissive, as with the high number of tests, we should correct for multiple testing. But when we corrected for multiple testing (Benjamini and Hochberg's method [29], same chance level), we found not any over-represented GO code for 43 of the 102 studies. With this cut-off, 85% of the kappa scores was 0 or lower and only 212 dataset pairs (4%) had a significant association at the 0.05 significance level. The poor overlap between datasets is partly caused by the fact that it is less likely to identify an over-represented GO code if the gene list is small.

### Reproduction of a manual clustering

**Figure 2:**
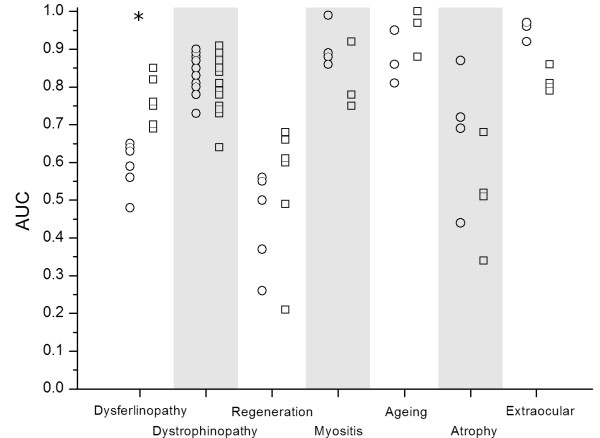
**Performance for reproduction of the manual grouping by kappa (circles) and LAMA (squares)**. A star indicates a statistically significant difference according to the Wilcoxon ranks test at the 0.05 level.

To evaluate the performance of the methods, we attempted to manually group the studies in the compendium based on similarities in the studied biological phenomena, before the start of the development of any new methodologies, We recognized 7 groups for a subset of 50 studies: dystrophin-deficiency (human and mouse), dysferlin-deficiency (human and mouse), myositis, regeneration and differentiation, ageing, atrophy, and extraocular muscle (EOM)-specific expression profiles (cf. the table in Additional file 1). A classification experiment was performed to evaluate to which extent the kappa and LAMA association scores could reproduce 7 manually identified clusters, using the area under the ROC curve (AUC) statistic. Results are shown in figure 2. The performance varies from near perfect scores for the ageing group to near random classification performance for the "regeneration and differentiation" and atrophy groups. Indeed, the latter groups studied more diverse conditions. The LAMA method performs better than the kappa for the dysferlinopathy, regeneration and ageing subgroups, though only for the dysferlinopathy group a statistically significant improvement is observed (p < 0.05; Wilcoxon rank test). Conversely, the kappa performed better for the myositis and the extraocular subgroups, but these differences are not statistically significant. Both methods performed similarly for the dystrophinopathy group.

The dysferlinopathy group has much higher classification rates with LAMA than in the kappa analysis. The studies in this group were more heterogeneous than the other groups in several aspects: it contained human and mouse studies, different mouse strains and differently aged mice, and four different microarray platforms were used in the six studies contained in this group. The human study that compared limb-girdle muscular dystrophy (LGMD) type 2B patients to controls (dataset 16) was much better classified to the dysferlinopathy group with the LAMA based approach than with the kappa based approach (AUC 0.73 vs 0.48). Datasets 16 (human) and 75a (dysferlin-deficient SJL mice versus controls) have no differentially expressed genes in common. Nevertheless, the LAMA score is the lowest possible score given the number of Monte Carlo simulations, which indicates there is a highly significant over-representation of gene associations. We identified "macrophage" as the most important shared concept between dysferlin-deficiency in humans and mice. Indeed, many of the identified associations are between genes known to be expressed in macrophages and macrophage infiltration is an important feature in both the LGMD patients pathology and the mouse model [30].

The slightly worse performance of the LAMA method in the classification of the myositis studies was due to strong associations with the group of dystrophinopathy studies. The two groups were found connected through concepts pertaining to inflammatory processes. This is reflective of the pronounced inflammatory component in both dystrophinopathies [21,22,31-33] and myositis patients. For the extraocular group the lower performance for LAMA is explained by the comparatively poor scores between datasets 4a and both 14a and b, while datasets 4b, 14a and 14b had high pairwise scores. Dataset 4a only contains 13 genes up-regulated genes, which limits the power for the LAMA analysis. The list shared only 2 genes with 14a and b, but still the kappa score was comparatively high due to the limited overlap overall. Classification for the GO-based over-representation analysis (p < 0.05, no correction for multiple testing) showed poor results: performance was considerably worse than based on gene list overlap for 5 of the 7 groups, a similar score was obtained for the dysferlinopathy group and a slightly better score for the ageing group. These results and a view on the shared GO codes indicated the used test condition was too lenient and spurious GO codes were assigned. Based on these results and the poor results presented in the previous section, we do not discuss the clustering based on the method here, but include the classification results and the clustering as supplementary material [see Additional file 3].

### Classification of new studies

To demonstrate the utility of our approach for the interpretation of gene lists from new experiments, we compared to our manual grouping the gene lists from a recent paper on dy/dy mice [34]. These mice have a muscular dystrophy as a consequence of a genetic defect in alpha-2 laminin. Our LAMA-approach classifies this study with high confidence in the dystrophinopathy group (AUC = 0.83). This is correct given the pathology of these mice and the two genetic deficiencies affecting the same macromolecular protein complex. The shared biological concepts between this dataset and a dataset from *mdx *mice (dataset 1; [22]), were the infiltration of macrophages and differential expression of collagens, metalloproteinases, cathepsins, and HLA-antigens.

### Dataset clustering based on kappa statistic

**Figure 3:**
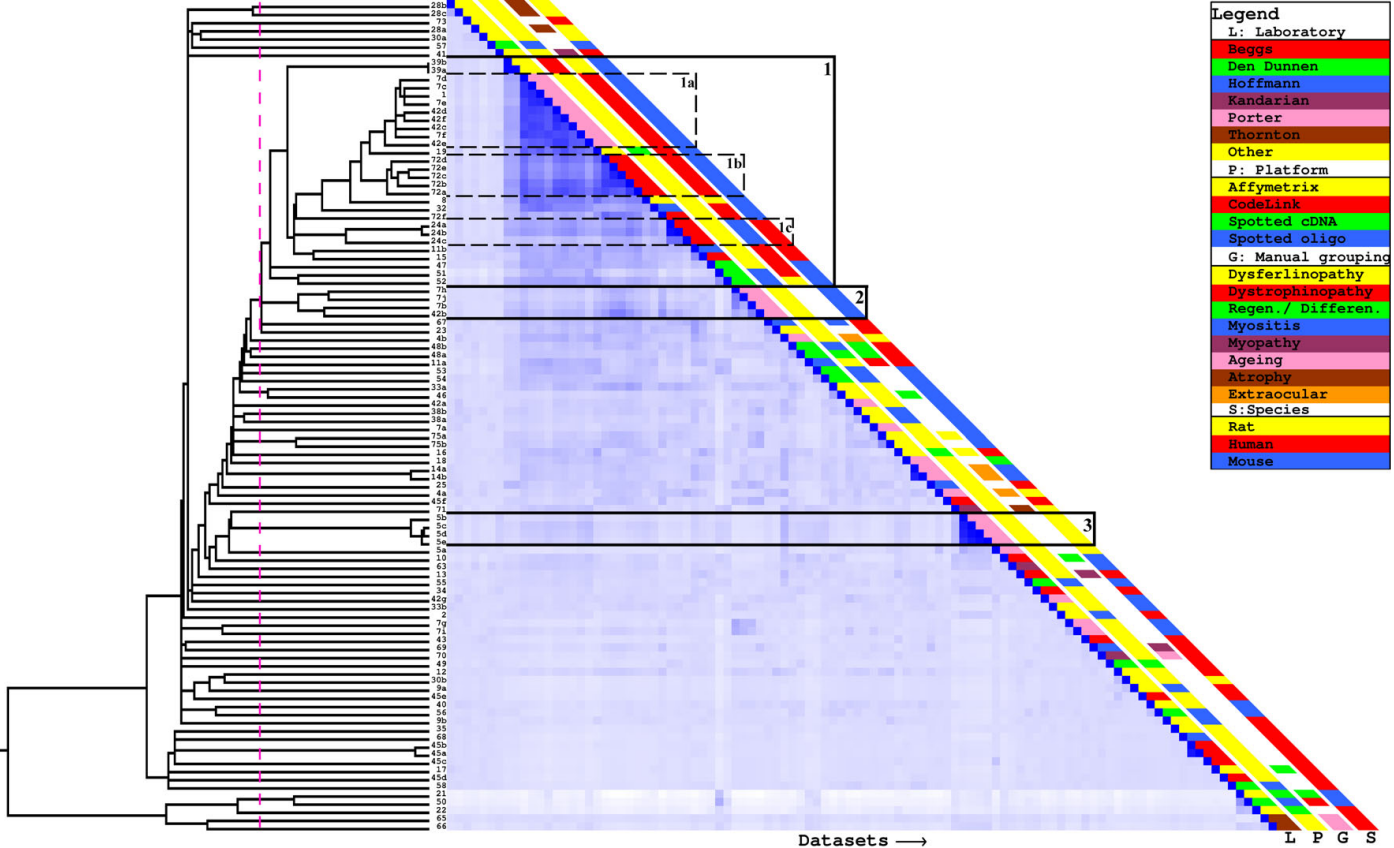
**Kappa-based hierarchical clustering and heatmap**. The dotted pink line indicates the used clustering cutoff and the identified clusters are indicated in addition to relevant subclusters. The dataset ids are shown between the tree and the heatmap. The colored bars provide background information on the datasets.

To get an overall view on the identified connections between studies, a hierarchical clustering of the microarray studies was performed using the kappa value as a similarity score (figure 3). One big cluster (indicated as cluster 1) and several smaller clusters were identified. Cluster 1 contains comparisons of the gene expression profiles between dystrophic subjects and healthy controls (dystrophin-deficient *mdx *mice (datasets 1, 7c-f, 42c-f, 19, 32 47, 51, 72a-f), dysferlin-deficient SJL mice (datasets 8, 39ab), patients with Duchenne muscular dystrophy (DMD, datasets 11b, 15)), as well as studies in human myositis patients (datasets 24a-c, cluster 1c). Similar to our note on the LAMA classification in the previous section, muscular dystrophy and myositis expression profiles have considerable overlap. Some muscular dystrophy studies unexpectedly fall outside cluster 1 and have only limited overlap to the datasets in cluster 1 (dataset 16, 67, 75a). We believe this to be at least partly attributable to technical factors. The color bars on the side of figure 3 illustrate that studies tend to cluster on microarray platform or laboratory. For example, the similar studies in the *mdx *mouse by Porter et al. (datasets 1, 7, and 42) and by Haslett et al. (dataset 72) do not cluster in a way that makes sense biologically, that is by age and muscle type-dependent severity of the disease. Instead they cluster by laboratory (cluster 1a – Porter; cluster 1b – Haslett).

Apart from the large dystrophy/myositis cluster of studies, there is only very limited overlap between the gene lists from the studies, as expected based on the pair-wise analysis presented above. Cluster 2 contains two studies investigating the spared EOM muscle in dystrophin-deficient *mdx *mice and the expression profiles of the diaphragm and hind limb muscles of presymptomatic *mdx *mice. Cluster 3 contains 4 highly overlapping studies (datasets 5b-d) from the same paper on developmental changes in the EOM muscle. Again, clusters 2 and 3 contain only studies done by the same group on the same platform.

### Dataset clustering based on LAMA

**Figure 4:**
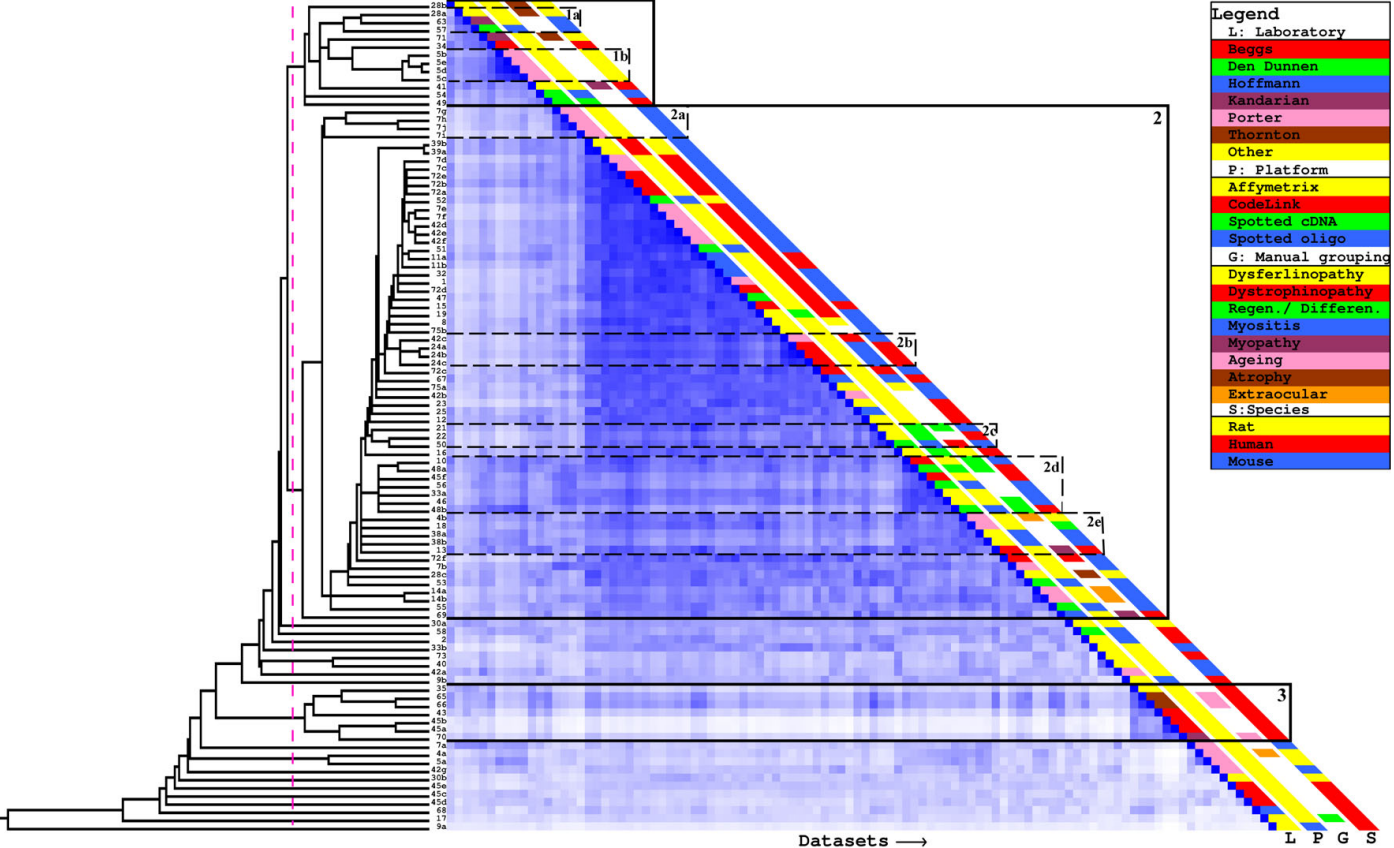
**LAMA-based hierarchical clustering and heatmap**. The dotted pink line indicates the used clustering cutoff and identified clusters are indicated in addition to relevat subclusters. The dataset ids are shown between the tree and the heatmap. The colored bars provide background information on the datasets.

The LAMA-based hierarchical clustering revealed more clusters and significant associations than the kappa-based clustering (figure 4). The side bars show that the LAMA-based clustering is less governed by technical factors like microarray platform and laboratory, and is better able to connect studies investigating the same biological phenomenon in different species or biological systems (cell culture or tissue; see below). Cluster 2 shows large overlap with cluster 1 in the kappa-based clustering, but contains many more studies. This cluster now contains all the studies on affected muscles in symptomatic *mdx *mice, all mouse models for LGMD, and all studies in human muscular dystrophy, including DMD, LGMD, and facioscapulohumeral dystrophy (FSHD), and myositis patients. The myositis patient profiles are closely associated with *mdx *mice of 23 days (dataset 42c) (subcluster 2b). We analysed the gene associations between dataset 24a (inclusion body myositis) and dataset 42c and found that the biological concept that contributes most to the associations is "chemokines". Indeed, at the analyzed age of 23 days the secretion of chemokines in the muscles of the *mdx *mice is maximal [22].

Table 1 shows the biological concepts underlying the gene associations in the different groups. For cluster 2, the most prominent biological terms for the upregulated genes were metalloproteinase activity (involved in extracellular matrix remodeling during fibrosis) and troponins. Metalloproteinases and troponins have been identified before to be important in muscular dystrophy (e.g. [22,35,36]) and in muscle regeneration. Studies on muscle regeneration are also included in cluster 2 (subcluster 2c). Remarkably, subcluster 2d contains all *in vitro *myoblast differentiation studies, both in primary human myoblast and in transformed mouse C2C12 myoblasts, whereas only a very small number of genes overlapped between the studies. As apparent from table 1, the concept "cullin proteins" formed the most significant link between the downregulated genes from studies on muscle regeneration (datasets 21, 22, 50). Since cullin proteins are ubiquitin ligases, it seems that ubiquitinylation is shut down during regeneration. This is an interesting discovery since ubiquitinylation activity was previously shown to be activated in the inverse condition, muscular atrophy [37,38].

**Table 1:** Characterizing concepts for clusters identified through LASSO analysis.

Cluster	Subcluster	Characteristic	Biological Concepts (Up)	Biological Concepts (Down)
**1**	**overall**	**Atrophy**	**-**	**Cyclins**
1	1A	Atrophy – PABPN1 overexpression	Amino acyl tRNA synthetases, spermidine, polyamines, spermine, eukaryotic initiation factors	Platelet-derived growth factor, transforming growth factor-beta, insulin-like growth factor binding proteins
1	1B	EOM-specific	Adipocytes, acyl CoA dehydrogenase	Cyclins, keratin, cyclin-dependent kinases
**2**	**overall**	**Dystrophy/myositis**	**Troponin, matrix metalloproteases**	**Mitogen activated protein kinases, insulin, ERK1 acitivity, phosphorylation**
2	2A	Dystrophin defiency in EOM muscle	Troponin	-
2	2B	Myositis	Chemokine, chemokine receptor	-
2	2C	Regeneration	T-lymphocyte, phosphotransferases, phosphorylation, mitogen-activated protein kinases, integrins, cell cycle	Cullin proteins, mitogen-activated protein kinases, ligase
2	2D	Differentiation	Troponin, tropomyosin, nemaline myopathies, sarcomeres, myosin heavy chain, calsequestrin	Inhibitor of differentiation proteins, E2F transcription factors, proteoglycan, cell cycle proteins
2	2E	Ky-mutant/diverse	Leptin, desaturase, myosin heavy chains, neural cell adhesion molecules	Mitogen-activated protein kinases
**3**	**overall**	**Ageing**	**Heterogeneous nuclear ribonucleoproteins, protein sumoylation, small nuclear ribonucleoprotein**	**-**

Concepts are shown separately for the down and up regulated gene lists. The column "Characteristic" gives a description of the studied phenomena in the cluster.

Cluster 1 was not found by the kappa-based clustering. Analysis of the underlying concept associations revealed similarities between the molecular processes during induced atrophy in cultured myoblasts (dataset 63) and *in vivo *models for muscular atrophy, i.e. during hind limb suspension or in space-flown rats (datasets 28a, 28b and 71). Amongst others, there is an interesting set of non-overlapping members of the semaphorin family shared between atrophy studies. Semaphorins are presumably involved in cell-cell contacts in neuronal cells [39] (during axon regeneration) but also in fusing myoblasts [40]. For cluster 1 we observe an increase in metabolic activity (both glycolytic and fatty acid oxidation) and a downregulation of extracellular matrix proteins: These processes seem to be revelant to age-related changes in EOM muscle (datasets 5b-d; subcluster 1b), and diverse myopathies mitochondrial encephelo myopathy (dataset 41), nemaline myopathy (dataset 34), and oculopharyngeal muscular dystrophy (OPMD; dataset 57). Cluster 3 contains all the ageing and sarcopenia studies. Interestingly, also a cell model for over-expression of the polyadenylation factor PABPN1 (also responsible for OPMD, dataset 35) is found in this cluster. In this case, the differences in RNA metabolism induced in the cell model aid the interpretation of the molecular phenotype observed in the ageing studies. Differences in RNA processing and splicing during ageing were also noted by the authors of the ageing studies [41,42].

## Discussion

The overlap between the gene lists of the microarray datasets in our compendium was limited, even though the studied phenomena were closely related. In addition, studies performed in the same laboratory or on the same microarray platform were more likely to demonstrate overlap than studies where more heterogeneous technologies and analysis approaches were used. The comparative analysis of the datasets through literature-derived gene associations resulted in the finding of many biologically relevant associations, and was more biology- than technology-driven. Both the analysis of the hierarchical clusterings and the reproduction of the manual clustering revealed that the LAMA method identified useful associations between datasets that were not retrieved by looking at gene overlap. Our method found these associations through correctly retrieved shared biological processes between the datasets.

Standard exploratory analysis based on an over-representation analysis of GO categories was not very powerful for our compendium, as shown by the lack of overlap between studies and the poor classification results. In general, the hypergeometric test will not often identify over-represented GO categories when short gene lists are analyzed. Yet, our association-based method was still able to find useful associations between datasets, even in cases where not a single shared over-represented GO code was found. Also, the associations we use cover a much broader range than GO. Indeed, not all of the concepts in table 1 are covered by the GO thesaurus (e.g. leptin). In addition, even if an appropriate GO term exists, it may not have been assigned any genes yet (e.g., cullin deneddylation).

Our broad network of associations increases our sensitivity for identifying interesting associations between datasets. We chose to use associations derived from literature to optimize for serendipity, but the network of associations could be taken from any source, including GO. An important feature of this approach is that by modulating the associations that are taken up in the network, the specificity and sensitivity of the found associations between datasets can be controlled.

Tomlins et al. [43] performed an over-representation analysis on gene lists representing the different stages of prostate cancer and used identified over-represented gene groups to compare the disease stages. The basis for their analysis was a database of 14000 groups of genes that share a relevant characteristic, or "molecular concept". They do not report low recall or lack of overlap of over-represented "molecular concepts". The likely explanation is that their meticulous sample preparation and highly standardized data generation and analysis avoided lack of overlap at the gene level and short gene lists. Clearly access to the raw data is commendable for exploratory studies, and standardized data generation is extremely useful given the high levels of variance observed with microarray experiments. It should be noted though that, besides the limited availability of raw data, the statistical models for the analysis of the raw data are hard to standardize. The choice for a statistical model depends on the design of the study, e.g. time course experiments or group comparisons, and technical factors, such as whether a one or two color microarray is used. Therefore perhaps the strongest point of our approach is that even with a wide range of study designs and statistical evaluations, across various platforms and species, a useful and insightful exploratory study was possible.

The issue of comparability between studies has been addressed for meta-analyses with a different objective than ours; the aggregation of information obtained from different DNA microarray studies [44-47]. It has been suggested that DNA microarray datasets could well be compared by the use of rankings of genes based on the level of significance of differential expression [47,48]. Indeed, also GSEA, a current popular method to test whether a set of genes is associated with the experimental variable is based on gene rankings [11]. It would be an interesting extension of the current work to use rankings in the dataset comparisons. A rank-based approach could be adapted to incorporate our text-based associations between genes. Also in this case, information on the statistical ranks is, however, frequently unavailable.

A limitation of the current LAMA analysis is that it relies on simulations to derive a measure to compare datasets. Simulations are computationally intensive, and have a resolution proportional to and limited by the number of performed iterations. The results presented here can be considered a proof of the utility of our approach, and a logical next step is to derive a model-based approximation as an alternative to our simulation-based measure. This would also avoid the necessity for the setting of a threshold on the gene association score. The currently applied threshold of 1% appears to be optimal [see Additional file 4]. When lowering the threshold, results will be more similar to the kappa method, as identity relations will start to dominate, whereas raising the threshold introduces noise and spurious connections.

## Conclusion

The compendium of studies showed limited overlap on gene ids, and a bias towards higher overlap between studies with technical similarities. The over-representation analysis based on GO categories was not very helpful in comparing studies, due to limited sensitivity and the incompleteness of the manually curated gene annotations. Compared to these approaches LAMA provided more biology- than technology-driven results and identified more biologically relevant associations between datasets. As the shared biological processes between studies could also be easily recognized, we believe LAMA is a powerful approach for the comparative meta-analysis of DNA microarray datasets.

## Methods

### Data acquisition

In our meta-analysis, we included DNA microarray studies on skeletal muscle development and/or disease. The compendium was limited to studies in human, mouse, and rat. Studies were included till December 2005. From each paper, lists of up- and downregulated genes were extracted from the tables reported in the paper or in the supplementary data. The compendium is not complete. For some of the studies, data could not be retrieved and requests for gene lists to the authors were unsuccessful. Since a full list of genes interrogated by each platform was essential for statistical analysis, studies on home-made arrays for which this information was not available had to be omitted as well. All probes on the array were mapped to Entrez Gene IDs. To be able to compare gene lists from the different organisms we mapped homologous genes to each other based on NCBI's HomoloGene database [20].

### Comparing DNA microarray experiments based on gene identity

The similarity between two datasets based on gene identity was measured using the kappa statistic [27]. Classically the kappa statistic is used to measure inter-rater reliability. It is more robust than simple agreement scores as it also takes agreement by chance into account. To use this measure the DNA microarray experiments are considered to assign every gene on the microarray platform a tag: upregulated, downregulated or the remainder category. The kappa statistic is defined as

$$\kappa =\frac{P(A)-P(E)}{1-P(E)}$$

where *P*(*A*) is the proportion of times that the two experiments give the same tag to a gene and *P*(*E*) is the expected proportion of times that the experiments give the same tag to a dataset. When calculating kappa we only consider the genes that are present on both platforms. If the DNA microarray datasets show identical results (*P*(*A*) = 1) then κ = 1. If the agreement is close to the level of agreement expected to occur by chance (*P*(*A*) ≈ *P*(*E*)) then κ ≈ 0.

### Recognizing references to concepts in texts

The corpus of literature for our experiments consisted of 3,160,002 MEDLINE abstracts, selected with the PubMed query "(protein OR gene) AND mammals". We used titles, MeSH headings, and abstracts. Stop words were removed and words were stemmed to their uninflected form by the LVG normalizer [49]. We used a thesaurus to identify concepts in texts. The thesaurus was composed of two parts: the 2006AC version of the UMLS thesaurus [50] and a gene thesaurus derived from multiple databases. The gene thesaurus was a combination of gene names from the rat genome database [51], mouse genome database [52], and a human gene thesaurus from several databases [53]. Homologous genes between the three species were mapped to each other using NCBI's HomoloGene database [20]. In order to exclude irrelevant concepts, two molecular biologists created a list of UMLS semantic types [see Additional file 5 for the complete list] relevant for biological information about genes. All concepts with other semantic types were removed from the thesaurus. Following Aronson [54], the UMLS thesaurus was also adapted for efficient natural language processing, avoiding overly ambiguous or duplicate terms, and terms that are very unlikely to be found in natural text. The gene thesaurus was expanded by rewrite rules to take into account common spelling variations [55]. For instance, numbers were replaced with roman numerals and vice versa, and hyphens before numbers at the end of gene symbols were inserted or removed (e.g. "WAF1" was rewritten as "WAF-1" and added as a synonym).

### Concept profile methodology

For every gene in our thesaurus that we identified in at least 5 documents, we characterized the documents in which the gene occurs with a concept profile. A concept profile of a concept *i*, for instance a gene, is an *M*-dimensional vector **w**_**i **_= (*w*_*i*1_, *w*_*i*2_, ⋯, *w*_*iM*_) where *M *is the number of concepts in the thesaurus. The weight *w*_*ij *_for a concept *j *in this profile indicates the strength of its association to the concept *i*. The weights in a concept profile for concept *i *are derived from the set of documents in which concept *i *occurs, *D*_*i*_, which is a subset of the total set of documents *D*.

To obtain the weight *w*_*ij *_we apply the symmetric uncertainty coefficient *U *(*X*_*i*_, *Y*_*j*_) [56] as suggested and evaluated earlier [57]:

$$w_{ij}=U(X_{i},Y_{j})=\frac{H(Y_{j})+H(X_{i})-H(X_{i},Y_{i})}{\frac{1}{2}(H(X_{i})+H(Y_{j}))}$$

Here the stochastic variable *X*_*i *_defines whether a document is in *D*_*i*_, and *Y*_*j *_gives the occurrence frequency of concept *j*. The entropies *H *are defined as follows:

$$\begin{array}{c} H(X_{i})=-\frac{O_{i}}{O}\ln\frac{O_{i}}{O}-\frac{O-O_{i}}{O}\ln\frac{O-O_{i}}{O},\\ H(Y_{j})=-\frac{o_{j}}{O}\ln\frac{o_{j}}{O}-\frac{O-o_{j}}{O}\ln\frac{O-o_{j}}{O},\\ H(X_{i},Y_{j})=-\frac{o_{ij}}{O}\ln\frac{o_{ij}}{O}-\frac{o_{j}-o_{ij}}{O}\ln\frac{o_{j}-o_{ij}}{O}-\frac{O_{i}-o_{ij}}{O}\ln\frac{O_{i}-o_{ij}}{O}-\frac{O-o_{j}-O_{i}+o_{ij}}{O}\ln\frac{O-o_{j}-O_{i}+o_{ij}}{O} \end{array}$$

where *O *and *O*_*i *_represent the number of concept occurrences in *D *and *D*_*i *_resp.; *o*_*j *_and *o*_*ij *_represent the number of occurrences of concept *j *in *D *and *D*_*i *_resp. The uncertainty coefficient is a normalized variant of the mutual information measure. The symmetric coefficient is the weighted average of the two assymmetric uncertainty coefficients: 1. the proportion of information in Y explained by knowledge of X and 2. the proportion of information in X explained by knowledge of Y.

### Literature-based comparison of gene lists

The similarity of the concept profiles of two genes was measured with the cosine similarity score [58]. If the similarity score exceeded a threshold, then the two genes were considered to have an association. For our experiments here, we calculated the similarity score for all pairs of genes for which a concept profile was available; the highest scoring 1% of pairs were taken as associations. A justification for the use of this threshold is given in the supplementary material. Subsequently, the associations are used to compare two gene lists. To do this, two gene lists were considered as separate sets of nodes, and the number of associations between the two were counted. We assessed how uncommon the observed number of associations was, by means of a distribution representing unassociated genelists. This distribution was estimated based on Monte Carlo simulations. For each simulation we performed the following two steps: 1. For each gene list we randomly selected a number of genes equal to the size of the gene list. These genes were selected from the genes present on the appropriate DNA microarray platform for which we had a concept profile available. 2. The number of connections between the two new gene lists were counted. Using the empirical distribution we subsequently estimated the chance of observing the given number of associations or more.

For each DNA microarray experiment we retrieved two gene lists, the upregulated and the downregulated genes. When comparing two experiments, p-values were computed for the two up and down lists; the final LAMA score was obtained by taking the log of the product of the two p-values. The log of the p-value product is commonly used in meta-analysis and is known as Fisher's method [59]. This score has been shown to follow a chi-square distribution, and can be used to derive a p-value. Here the measure is used to identify strongly associated datasets.

In order to interpret the LAMA score between two datasets we developed a computer program. The program shows for every gene in one set the associations that connect it to the other set. The biomedical concepts that underlie the gene associations could readily be retrieved and traced back to the literature through an incorporated version of Anni, a tool we published earlier [17]. To annotate a cluster of datasets we calculate the percentual contribution to the number of annotations for every gene, averaged over all dataset comparisons between cluster members. Subsequently we identified descriptive concepts for the cluster by retrieving concepts strongly associated to the top-ranking genes through the Anni annotation view. For table 1 we used as cutoffs 0.2% for selecting the genes and concepts in the annotation view were selected when their contribution was larger than 1. For brevity genes were excluded from this table. Of partially redundant concepts (e.g. "heterogeneous nuclear ribonucleoproteins" and "heterogeneous nuclear ribonucleoproteins activity") only the highest scoring concept was shown.

### High-throughput analysis of over-represented GO-terms

To evaluate if a set of differentially expressed genes shows an over-representation of genes belonging to a certain biological process, molecular function or cellular localization, as annotated by the Gene Ontology (GO) consortium [60], a hypergeometric test is commonly used, see e.g. the web tools DAVID [61] and GOTM [62]. We used the HyperGTest from the GOstats 2.0.4 package from the bioconductor open source software platform [63]. Only GO terms from the branch "Biological Process" subset of GO-terms were evaluated, since this was most relevant to the biological problem. To perform the test, an annotation package was built per species with the AnnBuilder 1.12.0 package in R, for the concatenated list of Entrez Gene identifiers represented by the relevant platforms. We analyzed up and down regulated gene lists separately. Similar to how we calculated the similarity of gene lists based on gene identifiers, the kappa statistic was used to calculate the similarity of significantly overrepresented GO-terms (p < 0.05) in the up-and downregulated gene sets from two microarray datasets.

### Reproduction of a manual clustering

We tested to which extent a manual grouping based on studied biological phenomena (cf. table 1) was reflected by the pair-wise similarity dataset scores by performing a classification experiment: The association measures were used to produce a ranking of the set of studies relative to one so-called seed study. All studies in turn served as a seed, producing a ranking for each of the other studies in the groups. Studies from the same group as the seed study were considered positive, studies from other groups negative cases. Based on the sorted list of positive and negative cases we constructed for each study a receiver operating characteristics (ROC) curve [64]. The area under the curve (AUC) was used as a performance measure [65]. An AUC of 1 represents perfect ordering, i.e. the studies from the same group as the selected study hold the top ranks, and an AUC of 0.5 is the expected score for a random ordering [65].

### Clustering DNA microarray data experiments

The DNA microarray studies can be compared to each other through the LAMA and kappa measures. To identify patterns in these associations we clustered the studies through agglomerative hierarchical clustering and subsequently annotated the identified clusters. For this purpose the LAMA scores were -*log*_10 _transformed.

## Abbreviations

AUC: The area under the receiver operating characteristics (ROC) curve; EOM: extraocular muscle; FSHD: facioscapulohumeral dystrophy; GO: gene ontology; LAMA: literature-aided meta-analysis; LGMD: limb-girdle muscular dystrophy; OPMD: oculopharyngeal muscular dystrophy.

## Authors' contributions

RJ developed the methodology, performed the experiments and wrote the manuscript. PACtH gathered the DNA microarray datasets, performed the biological evaluation of the results and wrote the manuscript. ES and JTdD aided in gathering the datasets and the data analysis. G–JBvO revised the manuscript. JAK supervised the development of the methodology and contributed to the manuscript. BM conceived of the study.

## Supplementary Material

Additional file 1 **Description of datasets**. Description of datasets in the compendium. The table gives for each dataset: 1. Pubmed ID (if available); 2. Paper first author; 3. Year of publication; 4. Species (human, mouse, rat); 5. Platform category; 6. Platform specification; 7. Studied material: tissue/cell-line; 8. Specification of tissue; 9. Studied condition; 10. Treatment; 11. Used control; 12. Used statistical test; 13. Number of up-regulated genes; 14. Number of down-regulated genes; 15. Grouping as performed by experts (see section). Click here for file

Additional file 2 **Compendium data**. The files containing the compendium data for the comparative meta-analysis. DatasetInfo.txt: This file defines the datasets and contains the entrez gene ids of the up and down regulated genes as well as some meta-info. Entries in this file are tab-separated and provides per dataset the following info: the dataset ID, species, a platform ID and on a newlines the the entrez gene IDs for the up and down regulated genes. PlatformInfo.txt: This file provides information about the platforms used for the experiments. The entrez gene ids of all the checked genes are included. Entries in this file are platformIDs (correspond to those mentioned in DatasetInfo) followed by tab separated Entrez gene IDs. Click here for file

Additional file 3 **Clustering and classification based on GO-overrepresentation analysis**. The file provides the classification results for the expert clustering as well as the hierarchically clustered heatmap of the studies according to a comparison of the identified overrepresented GO categories. Click here for file

Additional file 4 **Gene association cutoff for LAMA**. The concept profile association score is explored for the retrieval of gene associations. The overall distribution of the scores is shown and compared to the distributions of positive and negative gene associations. Click here for file

Additional file 5 **Semantic types selected for filtering**. The semantic filter applied for the comparison of concept profiles. Click here for file
