# Supplementary material for: Literature-aided meta-analysis of microarray data: a compendium study on muscle development and disease
Source: BMC Bioinformatics. 2008 Jun 24;9:291. doi: 10.1186/1471-2105-9-291 (PMC2459190; doi:10.1186/1471-2105-9-291)
Supplement: Additional file 1 — Description of datasets. Description of datasets in the compendium. The table gives for each dataset: 1. Pubmed ID (if available); 2. Paper first author; 3. Year of publication; 4. Species (human, mouse, rat); 5. Platform category; 6. Platform specification; 7. Studied material: tissue/cell-line; 8. Specification of tissue; 9. Studied condition; 10. Treatment; 11. Used control; 12. Used statistical test; 13. Number of up-regulated genes; 14. Number of down-regulated genes; 15. Grouping as performed by experts (see section). [file 1471-2105-9-291-S1.pdf]

Dataset	PubmedID	First author	Year	Species	Platform	Chip ID	Tissue / Cell line	Specification of tissue	Gene deficiency / condition	Treatment	Control / Reference	Cut-off used by authors	# Genes Up	# Genes Down	Expert group
1	11823445	Porter	2002	mouse	Affymetrix	U74A	skeletal muscle	gastrocnemius, quadriceps	dystrophin	no	wild-type age 8 wk	consistent diff. Wilcoxon's rank test, SAM, fold change values >=1.5	184	6	dystrophinopathy
2	15983191	Sparks	2005	human	Spotted oligonucl.	Home_Sparks	skeletal muscle	vastus lateralis	insulin-sensitive	high fat diet	insulin-sensitive males before high-fat diet	t-test p <0.01 (Bonferroni-adjusted)	94	110	
4a	12832294	Khanna	2003	rat	Affymetrix	U34A	skeletal muscle	extraocular	no	no	several muscles	fold change >2	13	0	extraocular
4b	12832294	Khanna	2003	rat	Affymetrix	U34A	skeletal muscle	extraocular	no	no	leg	consistent difference call, fold change >=2	93	54	extraocular
5a	15138310	Cheng	2004	rat	Affymetrix	U34A	skeletal muscle	extraocular	no	age 7d	timepoint 0 days (birth)	fold change >2	17	9	
5b	15138310	Cheng	2004	rat	Affymetrix	U34A	skeletal muscle	extraocular	no	age 14d	timepoint 0 days (birth)	fold change >2	113	64	
5c	15138310	Cheng	2004	rat	Affymetrix	U34A	skeletal muscle	extraocular	no	age 21d	timepoint 0 days (birth)	fold change >2	162	105	
5d	15138310	Cheng	2004	rat	Affymetrix	U34A	skeletal muscle	extraocular	no	age 28d	timepoint 0 days (birth)	fold change >2	142	149	
5e	15138310	Cheng	2004	rat	Affymetrix	U34A	skeletal muscle	extraocular	no	age 45d	timepoint 0 days (birth)	fold change >2	132	219	
7a	12874102	Porter	2003	mouse	Affymetrix	U74A	skeletal muscle	hindlimb	dystrophin	no	wild-type age 7d	consistent diff. from Wilcoxon's rank test, fold change values >=1.5	3	21	
7b	12874102	Porter	2003	mouse	Affymetrix	U74A	skeletal muscle	hindlimb	dystrophin	no	wild-type age 14d	consistent diff. from Wilcoxon's rank test, fold change values >=1.5	13	2	
7c	12874102	Porter	2003	mouse	Affymetrix	U74A	skeletal muscle	hindlimb	dystrophin	no	wild-type age 23d	consistent diff. from Wilcoxon's rank test, fold change values >=1.5	196	51	dystrophinopathy
7d	12874102	Porter	2003	mouse	Affymetrix	U74A	skeletal muscle	hindlimb	dystrophin	no	wild-type age 28d	consistent diff. from Wilcoxon's rank test, fold change values >=1.5	108	21	dystrophinopathy
7e	12874102	Porter	2003	mouse	Affymetrix	U74A	skeletal muscle	hindlimb	dystrophin	no	wild-type age 56d	consistent diff. from Wilcoxon's rank test, fold change values >=1.5	362	65	dystrophinopathy
7f	12874102	Porter	2003	mouse	Affymetrix	U74A	skeletal muscle	hindlimb	dystrophin	no	wild-type age 112d	consistent diff. from Wilcoxon's rank test, fold change values >=1.5	99	73	dystrophinopathy
7g	12874102	Porter	2003	mouse	Affymetrix	U74A	skeletal muscle	extraocular	dystrophin	no	wild-type age 14d	consistent diff. from Wilcoxon's rank test, fold change values >=1.5	3	2	
7h	12874102	Porter	2003	mouse	Affymetrix	U74A	skeletal muscle	extraocular	dystrophin	no	wild-type age 28d	consistent diff. from Wilcoxon's rank test, fold change values >=1.5	7	3	
7i	12874102	Porter	2003	mouse	Affymetrix	U74A	skeletal muscle	extraocular	dystrophin	no	wild-type age 56d	consistent diff. from Wilcoxon's rank test, fold change values >=1.5	5	16	
7j	12874102	Porter	2003	mouse	Affymetrix	U74A	skeletal muscle	extraocular	dystrophin	no	wild-type age 112d	consistent diff. from Wilcoxon's rank test, fold change values >=1.5	16	3	
8	14506282	Lennon	2003	mouse	Affymetrix	U74A	skeletal muscle	gastrocnemius, quadriceps	dysferlin	no	wild-type age 6 wk and 8 months	Correlation networks	74	107	dysferlinopathy
9a	15687108	Teran-Garcia	2005	human	Spotted oligonucl.	Home_Pennington	skeletal muscle	unknown	no insulin sensi. change (bef. training)	exercise	insulin sensitivity improvement (bef. training)	fold change >1.4	21	4	
9b	15687108	Teran-Garcia	2005	human	Spotted oligonucl.	Home_Pennington	skeletal muscle	unknown	no insulin sensi. change (posttraining)	exercise	insulin sensitivity improvement (posttraining)	fold change >1.4	29	28	
10	14688207	Tomczak	2004	mouse	Affymetrix	U74A+C	myoblast	C2C12	no	differentiation	undifferentiated	CAGED clustering, fold-change >2.0 relative to time -2 days	448	352	regen. and diff.
11a	11121445	Chen	2000	human	Affymetrix	HuGeneFL	skeletal muscle	unknown	dystrophin	no	healthy	Rosetta error model p <0.01 (Benjamini-Hochberg corrected)	341	161	dystrophinopathy
11b	11121445	Chen	2000	human	Affymetrix	HuGeneFL	skeletal muscle	unknown	dystrophin	no	healthy	fold-change >=2	41	39	dystrophinopathy
12	14519683	Winokur	2003	human	Affymetrix	HuGeneFL + U95A	skeletal muscle	biceps	FSHD	no	healthy	fold-change >2 on U95Av2 and HuFL	13	10	
13	12677001	Sanoudou	2003	human	Affymetrix	U95A+B	skeletal muscle	unknown	nemaline myopathy	no	healthy	t-test p <0.001	58	115	myopathy
14a	11572940	Porter	2001	mouse	Affymetrix	U74A	skeletal muscle	extraocular	no	no	gastrocnemius and soleus muscle	SAM FDR <=0.4	24	22	extraocular
14b	11572940	Porter	2001	mouse	Affymetrix	U74A	skeletal muscle	extraocular	no	no	jaw	SAM FDR <=0.4	30	36	extraocular
15	12415109	Haslett	2002	human	Affymetrix	U95A	skeletal muscle	quadriceps	dystrophin	no	healthy	t-test p < 0.0001; FDR = 0.0023	70	14	dystrophinopathy
16	12471055	Campanaro	2002	human	Spotted cDNA	Home_muscle_Bologna	skeletal muscle	unknown	dysferlin	no	healthy	fold-change >=1.8	43	13	dysferlinopathy
17	14519196	Zambon	2003	human	Affymetrix	U95A	skeletal muscle	vastus lateralis	no	exercise	leg	paired t-test p <0.05 at 6 and 18h	8	8	regen. and diff.
18	15326121	Khanna	2004	monkey	Affymetrix	U133A	skeletal muscle	Orbital layer of ocular musle	no	no	global layer of ocular muscle	t-test p <0.05 (RMA and MAS), fold-change>=2	71	62	
19	12176734	Rouger	2002	mouse	Spotted cDNA	Home_INSERM_DD	skeletal muscle	hindlimb	dystrophin	no	healthy	fold change >2 at different time points	23	11	dystrophinopathy
21	12477723	Yan	2003	mouse	Spotted cDNA	Home_NIH_Caltech	skeletal muscle	tibialis anterior	no	Cardiotoxin	untreated	fold change >2.5	1176	1004	regen. and diff.
22	15126512	Viguerie	2004	human	Spotted cDNA	Home_Viguerie	skeletal muscle	vastus lateralis	no	epinephrine	healthy	SAM FDR <0.001	1107	402	
23	15598661	Richardson	2005	human	Affymetrix	U133A	skeletal muscle	vastus lateralis	no	lipid infusion	saline	SAM and Genespring	26	0	
24a	12391344	Greenberg	2002	human	Affymetrix	U95A	skeletal muscle	unknown	inclusion body myositis	no	healthy	fold-change >=10 minexpr=500	125	0	myositis
24b	12391344	Greenberg	2002	human	Affymetrix	U95A	skeletal muscle	unknown	polymyositis	no	healthy	fold-change >=10 minexpr=500	121	0	myositis
24c	12391344	Greenberg	2002	human	Affymetrix	U95A	skeletal muscle	unknown	dermatomyositis	no	healthy	fold-change >=10 minexpr=500	45	0	myositis
25	12937035	Chen	2003	human	Affymetrix	U95A	skeletal muscle	vastus lateralis	no	ec-&concentric contraction	concentric contractions only	t-test p <0.05, fold-change >5	24	0	
28a	14715702	Nikawa	2004	rat	Affymetrix	U34A+B+C	skeletal muscle	gastrocnemius	no	space flight	untreated	fold change >=8	59	101	atrophy
28b	14715702	Nikawa	2004	rat	Affymetrix	U34A+B+C	skeletal muscle	gastrocnemius	no	tail suspension	untreated	fold change >=8	22	18	atrophy
28c	14715702	Nikawa	2004	rat	Affymetrix	U34A+B+C	skeletal muscle	gastrocnemius	no	denervation	untreated	fold change >=8	67	29	atrophy
30a	14618091	Komamura	2003	rat	Affymetrix	U34A	skeletal muscle	gastrocnemius	no	dexamethasone	untreated	fold change >=3	6	9	
30b	14618091	Komamura	2003	rat	Affymetrix	U34A	skeletal muscle	gastrocnemius	no	dexamethasone+IGF	untreated	fold change >=3	6	1	
32	12133862	Tseng	2002	mouse	Affymetrix	U74A	skeletal muscle	gastrocnemius	dystrophin	no	wild-type age 16 wk	t-test p <0.01 (Benjamini-Hochberg corrected); fold-change >=2	97	16	dystrophinopathy
33a	15475267	Kuninger	2004	mouse	Affymetrix	U74A+B+C	myoblast	C2AS12	no	IGF	untreated	fold change >=3	67	0	
33b	15475267	Kuninger	2004	mouse	Affymetrix	U74A+B+C	myoblast	C2AS12	no	PDGF	untreated	fold change >=3	43	0	
34	15056467	Sanoudou	2004	human	Affymetrix	U95A+B	skeletal muscle	unknown	nemaline myopathy non-typing	no	nemaline myopathy typing	correlation method	182	76	
35	15755682	Corbeil-Girard	2005	human	Affymetrix	U133A	lung carcinoma cells	A549tTA	Ala expansion in overexpr.Pabpn1	no	mock-infected	fold change >=2.5; Intensity difference > 200 units	88	87	
38a	15036332	Blanco	2004	mouse	Spotted oligonucl.	Home_Compugen_Brown	skeletal muscle	soleus (affected)	unknown - ky	no	wild-type	Bayes probability; additional cut-off for signal units.	19	54	
38b	15036332	Blanco	2004	mouse	Spotted oligonucl.	Home_Compugen_Brown	skeletal muscle	EDL (unaffected)	unknown - ky	no	wild-type	Bayes probability; additional cut-off for signal units.	15	15	
39a	15811552	Suzuki	2005	mouse	Codelink	Uniset mouse I	skeletal muscle	quadriceps	dysferlin	no	wild-type	t-test p <0.05, fold change >2	115	59	dysferlinopathy
39b	15811552	Suzuki	2005	mouse	Codelink	Uniset mouse I	skeletal muscle	quadriceps	dysferlin	no	wild-type	t-test p <0.05, fold change >2	119	79	dysferlinopathy
40	15272020	Kamei	2004	mouse	Affymetrix	U74A	skeletal muscle	quadriceps	FOXO1	no	wild-type	Fisher's protected least significant difference test	0	18	
41	15728662	Crimi	2005	human	Affymetrix	U133A	skeletal muscle	unknown	mitochondrial encephalo-myopathy	no	healthy	fold change >=2.5	43	59	myopathy
42a	14681298	Porter	2004	mouse	Affymetrix	U74A	skeletal muscle	diaphragm	dystrophin	no	wildtype - age 7d	pairwise replicate comparisons Wilcoxon p <0.01	1	33	
42b	14681298	Porter	2004	mouse	Affymetrix	U74A	skeletal muscle	diaphragm	dystrophin	no	wildtype - age 14d	pairwise replicate comparisons Wilcoxon p <0.01	9	4	
42c	14681298	Porter	2004	mouse	Affymetrix	U74A	skeletal muscle	diaphragm	dystrophin	no	wildtype - age 23d	pairwise replicate comparisons Wilcoxon p <0.01	192	26	dystrophinopathy
42d	14681298	Porter	2004	mouse	Affymetrix	U74A	skeletal muscle	diaphragm	dystrophin	no	wildtype - age 28d	pairwise replicate comparisons Wilcoxon p <0.01	185	43	dystrophinopathy
42e	14681298	Porter	2004	mouse	Affymetrix	U74A	skeletal muscle	diaphragm	dystrophin	no	wild-type - age 56d	pairwise replicate comparisons Wilcoxon p <0.01	234	213	dystrophinopathy
42f	14681298	Porter	2004	mouse	Affymetrix	U74A	skeletal muscle	diaphragm	dystrophin	no	wild-type - age 112 d	pairwise replicate comparisons Wilcoxon p <0.01	144	51	dystrophinopathy
42g	14681298	Porter	2004	mouse	Affymetrix	U74A	skeletal muscle	diaphragm	no	no	hindlimb	pairwise replicate comparisons Wilcoxon p <0.01	28	11	
43	14625377	Sanoudou	2004	human	Affymetrix	U95A	skeletal muscle	quadriceps, others	no	autopsy	surgical	SAM FDR 0.0166, fold-change >2	119	0	
45a	15962335	Kang	2005	human	Affymetrix	U133A	skeletal muscle	deltoid	no	no	quadriceps	PCA	65	0	
45b	15962335	Kang	2005	human	Affymetrix	U133A	skeletal muscle	deltoid	no	no	gastrocnemius	PCA	65	0	
45c	15962335	Kang	2005	human	Affymetrix	U133A	skeletal muscle	deltoid	no	no	tibialis anterior	PCA	4	0	
45d	15962335	Kang	2005	human	Affymetrix	U133A	skeletal muscle	gastrocnemius	no	no	quadriceps	PCA	18	0	
45e	15962335	Kang	2005	human	Affymetrix	U133A	skeletal muscle	gastrocnemius	no	no	tibialis anterior	PCA	6	0	
45f	15962335	Kang	2005	human	Affymetrix	U133A	skeletal muscle	quadriceps	no	no	tibialis anterior	PCA	37	0	
46	12837262	Delgado	2003	mouse	Affymetrix	U74A	myoblast	C2C12	no	differentiation	undifferentiated	K-means clustering; fold change >3.5	102	32	regen. and diff.
47	12206806	Boer	2002	mouse	Affymetrix	U74A	skeletal muscle	hindlimb	dystrophin and dystrophin x utrophin	no	wild-type	t-test p< 0.01	79	60	dystrophinopathy
48a	15336692	Sterrenburg	2004	human	Spotted cDNA	LGTC_muscle	myoblast	primary	no	differentiation	undifferentiated	Z > 1.96	68	63	regen. and diff.
48b	15336692	Sterrenburg	2004	human	Spotted oligonucl.	LGTC_Hu19K	myoblast	primary	no	differentiation	undifferentiated	ANOVA p <0.000005; Fold-change >2	59	59	regen. and diff.
49	16679024	Sterrenburg	2005	human	Spotted cDNA	LGTC_muscle	myoblast	primary	dystrophin	differentiation	undifferentiated, healthy	ANOVA p <0.00005	39	46	
50	16011810	Turk	2005	mouse	Spotted oligonucl.	LGTC_Mu7.5K	skeletal muscle	quadriceps	dystrophin	no	healthy	p <1e-6 at least one time point	717	812	dystrophinopathy
51	16306063	Turk	2005	mouse	Spotted oligonucl.	LGTC_Mu22K	skeletal muscle	quadriceps	different severe muscular dystrophies	no	healthy and less affected animals	ANOVA p <0.05 (Benjamini-Hochberg corrected)	518	482	dystrophinopathy
52	16306063	Turk	2005	mouse	Spotted oligonucl.	LGTC_Mu22K	skeletal muscle	quadriceps	dysferlin	no	healthy	ANOVA p <0.05 (Benjamini-Hochberg corrected)	148	157	dysferlinopathy
53	NA	't Hoen	2005	mouse	Spotted oligonucl.	LGTC_Mu7.5K	skeletal muscle	quadriceps	beta-sarcoglycan	no	healthy	p<0.05 temporal expression profile: hotelling t-test	268	159	
54	NA	't Hoen	2005	mouse	Spotted oligonucl.	LGTC_Mu7.5K	skeletal muscle	quadriceps	gamma-sarcoglycan	no	healthy	p<0.05 temporal expression profile: hotelling t-test	51	63	
55	NA	Duguez	2005	mouse	Spotted oligonucl.	LGTC_Mu22K	skeletal muscle	psoas and quadriceps	calpain3	no	wild-type	Rosetta error model p <0.05 (Benjamini-Hochberg corrected)	134	126	
56	NA	't Hoen	2005	mouse	Spotted oligonucl.	LGTC_Mu22K	skeletal muscle	quadriceps	fxr	no	healthy	ANOVA p <0.05 (Benjamini-Hochberg corrected) in female and male	56	12	
57	NA	Routledge	2005	mouse	Spotted oligonucl.	LGTC_Mu22K	myoblast	immortalized IM2	Ala expansion in overexpr. Pabpn1	no	untransfected	ANOVA p <0.05 Bonf. corr.; Similarity (PABPN1) -0.9 > p > 0.9	30	74	
58	NA	Sterrenburg	2005	human	Spotted oligonucl.	LGTC_Hu19K	skeletal muscle	quadriceps	Ala expansion in PABPN1 gene	no	healthy	ANOVA p <0.05 Bonferroni corrected	43	18	
63	15608089	Stevenson	2005	mouse	Affymetrix	MOE430A	myotube	C2C12	no	starvation / atrophy	untreated	fold-change >=2	72	268	
65	12783983	Welle	2003	human	Affymetrix	U133A+B	skeletal muscle	vastus lateralis	no	ageing	young men	t-test p <0.05; SAM FDR <0.1	213	191	ageing
66	15036396	Welle	2004	human	Affymetrix	U133A+B	skeletal muscle	vastus lateralis	no	ageing	young women	t-test p <0.01; SAM FDR <0.1	432	491	ageing
67	11937576	Tezak	2002	human	Affymetrix	HuGeneFL	skeletal muscle	unknown	juvenile dermatomyositis	no	healthy	fold-change >=2 (pairwise comparison)	37	29	myositis
68	15079007	Molon	2004	human	Affymetrix	U95A+U133A	skeletal muscle	vastus lateralis	spastic paraplegia	no	healthy	Welch t-test p <0.05; fold-change >=2 on U95+U133	0	27	
69	14755723	Di Giovanni	2004	human	Affymetrix	U95A	skeletal muscle	unknown	acute quadriplegic myopathy	no	healthy	Welch ANOVA t-test p <0.05; fold-change >=1.5	20	0	myopathy
70	15687482	Giresi	2005	human	Affymetrix	U133A	skeletal muscle	vastus lateralis	no	sarcopenia in older subjects	young control subjects	t-test p <0.01	26	18	ageing
71	12844509	Stevenson	2003	rat	Affymetrix	U34A	skeletal muscle	hindlimb	no	disuse (atrophy)	untreated	ANOVA p <0.0538 Benjamini-Hochberg-corrected	218	95	atrophy
72a	16261416	Haslett	2005	mouse	Affymetrix	U74A	skeletal muscle	diaphragm	dystrophin	no	wild-type age 8 wk	Geometric fold change analysis	99	42	dystrophinopathy
72b	16261416	Haslett	2005	mouse	Affymetrix	U74A	skeletal muscle	EDL	dystrophin	no	wild-type age 8 wk	Geometric fold change analysis	69	3	dystrophinopathy
72c	16261416	Haslett	2005	mouse	Affymetrix	U74A	skeletal muscle	gastrocnemius	dystrophin	no	wild-type age 8 wk	Geometric fold change analysis	30	1	dystrophinopathy
72d	16261416	Haslett	2005	mouse	Affymetrix	U74A	skeletal muscle	quadriceps	dystrophin	no	wild-type age 8 wk	Geometric fold change analysis	39	43	dystrophinopathy
72e	16261416	Haslett	2005	mouse	Affymetrix	U74A	skeletal muscle	soleus	dystrophin	no	wild-type age 8 wk	Geometric fold change analysis	31	8	dystrophinopathy
72f	16261416	Haslett	2005	mouse	Affymetrix	U74A	skeletal muscle	tibialis anterior	dystrophin	no	wild-type age 8 wk	Geometric fold change analysis	12	15	dystrophinopathy
73	16263771	Balagopal	2005	human	Affymetrix	U133A+B	skeletal muscle	unknown	dystrophin	oxandrolone	untreated DMD patients	fold-change >2	10	67	
75a	16288871	Von der Hagen	2005	mouse	Affymetrix	U74A+B+C	skeletal muscle	quadriceps, tibialis anterior	dysferlin	no	wild-type age 21d	t-test p <0.1; fold-change >2	27	15	dysferlinopathy
75b	16288871	Von der Hagen	2005	mouse	Affymetrix	U74A+B+C	skeletal muscle	quadriceps	no	no	tibialis anterior	t-test p <0.1; fold-change >2	30	12	
