# Supplementary material for: Literature-aided meta-analysis of microarray data: a compendium study on muscle development and disease
Source: BMC Bioinformatics. 2008 Jun 24;9:291. doi: 10.1186/1471-2105-9-291 (PMC2459190; doi:10.1186/1471-2105-9-291)

Appendix: Clustering and classification based on GO-overrepresentation analysis.

Table 1: Median classification scores (AUC) for the kappa and GO overrepresentation measure ($p<0.05$)

Group	kappa	GO
Dysferlinopathy	0.61	0.6
Dystrophinopathy	0.85	0.75
Regeneration	0.53	0.5
Myositis	0.89	0.85
Ageing	0.86	0.96
Atrophy	0.71	0.44
Extraocular	0.97	0.51

Figure 1: Clustering based on GO overrepresentation analysis ($p<0.05$).

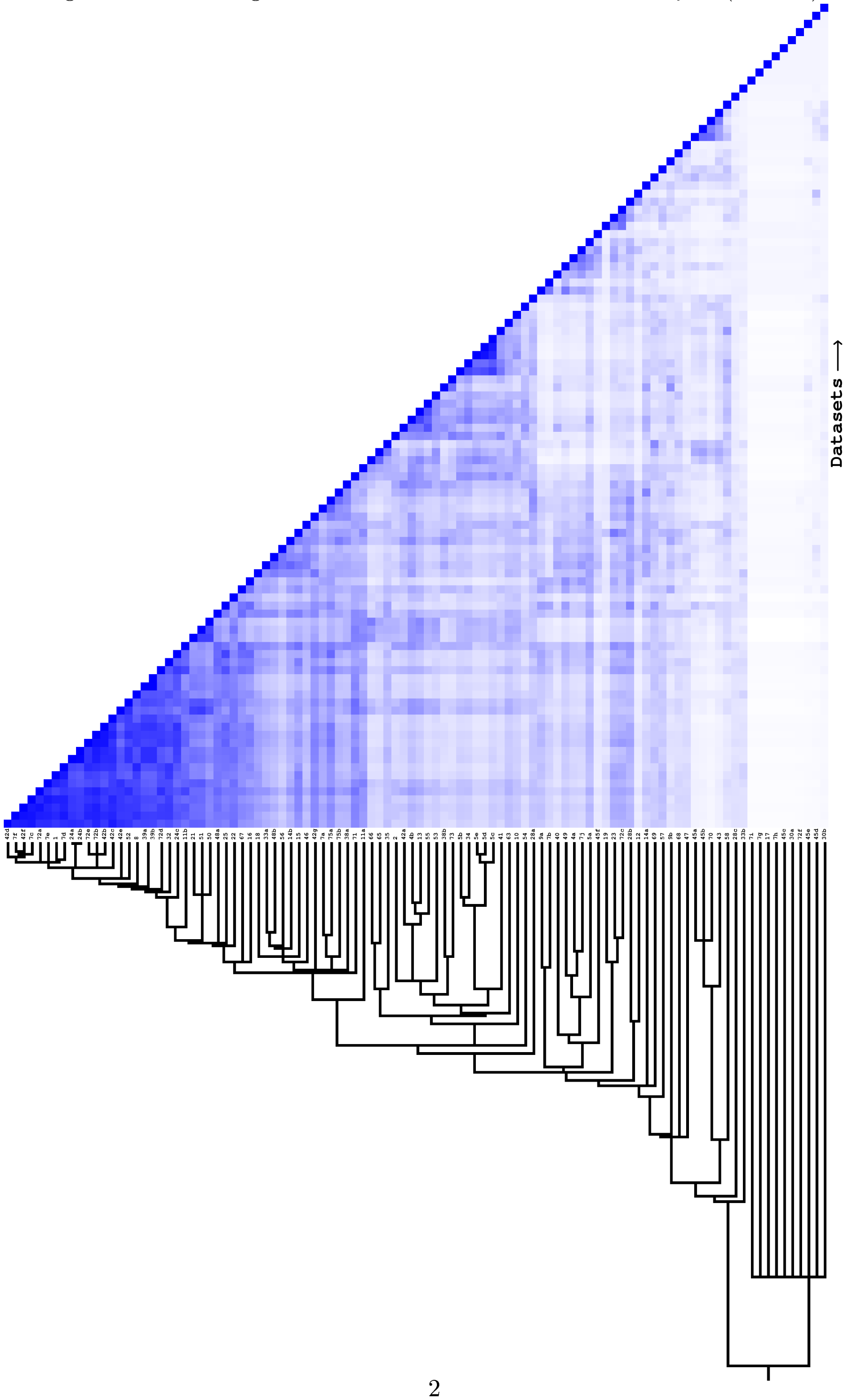

Supplement: Additional file 3 — Clustering and classification based on GO-overrepresentation analysis. The file provides the classification results for the expert clustering as well as the hierarchically clustered heatmap of the studies according to a comparison of the identified overrepresented GO categories. [file 1471-2105-9-291-S3.pdf]
