# Supplementary material for: Literature-aided meta-analysis of microarray data: a compendium study on muscle development and disease
Source: BMC Bioinformatics. 2008 Jun 24;9:291. doi: 10.1186/1471-2105-9-291 (PMC2459190; doi:10.1186/1471-2105-9-291)
Supplement: Additional file 4 — Gene association cutoff for LAMA. The concept profile association score is explored for the retrieval of gene associations. The overall distribution of the scores is shown and compared to the distributions of positive and negative gene associations. [file 1471-2105-9-291-S4.pdf]

Appendix: Gene Association Cutoff for LASSO

A cutoff is used on the gene association scores retrieved with concept profiles to identify relevant associations. This cutoff may be an important variable for the performance of the LASSO system. In this appendix the distribution of the gene association scores is evaluated. In the figure below the distribution is compared to random association scores and positive associations. The random associations can be taken as an indication for the level of noise, or false associations. For the randomization concept profiles were generated based on random literature selections. In these concept profiles a concept was added to represent the gene that would have defined the document selection and that was taken to occur on average twice in every document in the set. A set of positive associations was also included to get an idea of fraction of true positive associations that fall above the threshold, and hence the recall of positive associations. As an appo-raximation of a set of positives, gene pairs with at least ten co-occurrences in literature were taken. As can be seen the chosen threshold of 1% excludes most of the negative associations and captures the majority of positive associations. This is further illustrated in the accompanying table, showing the percentages of included associations for various cutoff values.

Figure 1: Distributions for the (log transformed) gene associaton scores (black), randomized scores (red) and scores for the positive gene associations (blue). The vertical line indicates the 1% threshold.

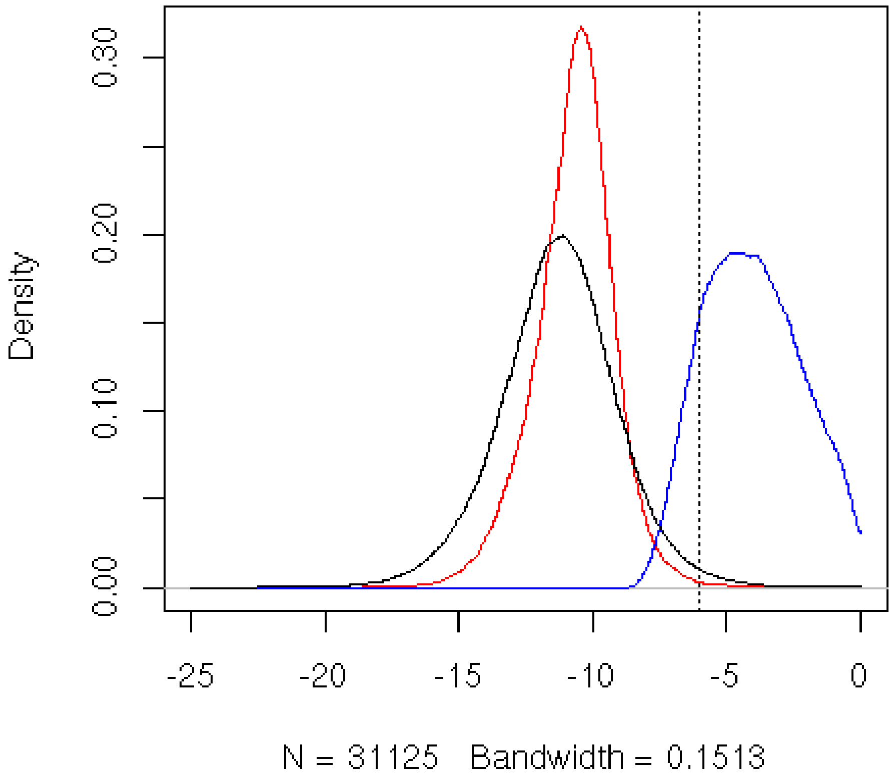

Table 1: The percentages of included associations for positive and negative associations for different percentile cutoff values for the gene associations.

cutoff (percentile)	%included pos	%included neg
5	100	2.4
1	85	0.28
0.50	66	0.08
0.10	31	0.02
0.05	21	0.01
